# Pre-Frailty and Frailty in Hospitalized Older Adults: A Comparison Study in People with and without a History of Cancer in an Acute Medical Unit

**DOI:** 10.3390/cancers16122212

**Published:** 2024-06-13

**Authors:** Chad Yixian Han, Raymond Javan Chan, Huah Shin Ng, Yogesh Sharma, Alison Yaxley, Claire Baldwin, Michelle Miller

**Affiliations:** 1 Caring Futures Institute, College of Nursing and Health Sciences, Flinders University, Adelaide, SA 5042, Australia; raymond.chan@flinders.edu.au (R.J.C.); alison.yaxley@flinders.edu.au (A.Y.); claire.baldwin@flinders.edu.au (C.B.); michelle.miller@flinders.edu.au (M.M.); 2 Faculty of Health, Queensland University of Technology, Brisbane, QLD 4000, Australia; 3 College of Medicine and Public Health, Flinders University, Adelaide, SA 5042, Australia; huahshin.ng@flinders.edu.au (H.S.N.); yogesh.sharma@sa.gov.au (Y.S.); 4 SA Pharmacy, SA Health, Adelaide, SA 5001, Australia; 5 Department of Acute and General Medicine, Flinders Medical Centre, Adelaide, SA 5042, Australia

**Keywords:** older adults, frailty, pre-frailty, cancer, hospitalized, acute medical unit

## Abstract

**Simple Summary:**

A prospective observational study was conducted in a cohort of 329 hospitalized older adults ≥65 years admitted to the acute medical unit (AMU) of a tertiary hospital. Statistical models compared older adults with and without a history of cancer to determine characteristics associated with pre-frailty/frailty and length of hospital stay (LOS). About one-fifth of the cohort had a history of cancer. The prevalence of pre-frailty/frailty among older adults with a history of cancer was 58%. In patients with a history of cancer, pre-frailty/frailty was associated with eight and nine times more likelihood of experiencing polypharmacy and malnutrition, respectively. The risk of having a longer LOS was 24% higher in older adults with a history of cancer than those without. Further investigations are warranted for improvement to systematic assessments to identify those at risk and provide interventions to meet the complex needs of this vulnerable population.

**Abstract:**

A prospective observational study was conducted in a cohort of older adults ≥65 years (*n* = 329), admitted to the acute medical unit (AMU) of a tertiary hospital, to describe and compare characteristics including frailty status and clinical outcomes. Multivariable models compared older adults with and without a history of cancer to determine characteristics associated with frailty and pre-frailty. An adjusted Poisson regression model was used to compare the length of hospital stay (LOS) between the two groups. About one-fifth (22%) of the cohort had a history of cancer. The most common cancer types were prostate (*n* = 20), breast (*n* = 13), lung (*n* = 8) and gastrointestinal (*n* = 8). There was no difference in the prevalence of pre-frailty/frailty among patients with or without a history of cancer (58% vs. 57%, *p* > 0.05). Pre-frailty/frailty was associated with polypharmacy (OR 8.26, 95% CI: 1.74 to 39.2) and malnutrition (OR 8.91, 95% CI: 2.15 to 36.9) in patients with a history of cancer. Adjusted analysis revealed that the risk of having a longer LOS was 24% higher in older adults with a history of cancer than those without (IRR 1.24, 95% CI 1.10 to 1.41, *p* < 0.001). Clinicians in the AMU should be aware that older adults with a history of cancer have a higher risk of a longer LOS compared to those without.

## 1. Introduction

Frailty and pre-frailty can significantly impact older adults who are admitted to hospital, influencing their treatment outcomes, quality of life, healthcare utilization and mortality rates [1,2]. This is of particular importance for those with a history of cancer. Older adults make up a significant percentage of people with cancer and cancer deaths [3]. The prevalence of pre-frailty and frailty vary across settings, i.e., community (ambulatory and population-based), nursing home and hospital; with the highest prevalence of pre-frailty reported in hospital settings [4]. The incidence of frailty is particularly high among older adults with cancer, considering that the disease itself and its treatments are stressors that significantly tax their physiologic reserve [5]. Previous research has demonstrated that older adults with cancer who are frail have decreased chemotherapy tolerance, increased risk of postoperative complications and disease progression, and mortality [6].

The Fried frailty criteria phenotype model and the Rockwood frailty deficit-accumulation model are two generally recognized models/theories underpinning instruments used to identify frailty and pre-frailty in older adults [7]. There are many instruments developed to identify frailty and pre-frailty, and the Edmonton Frail Scale (EFS) is one of the most commonly used pre-frailty and frailty assessment tools for hospitalized older adults [8,9].

Although many frailty-specific screening and assessment tools have been developed, with a subset of them also validated for the oncology population, there continues to be a lack of knowledge and their implementation amongst oncological healthcare professionals worldwide [10,11]. At present, there is no consensus on the optimum frailty screening or assessment tool for the geriatric oncology population [12]. In fact, frailty and pre-frailty can also be detected using non-frailty-specific assessment tools used in oncology populations (e.g., the scored patient-generated subjective global assessment (PG-SGA)), which is primarily a nutritional assessment tool [13]. While there is significant variability in how studies categorize frailty and pre-frailty within the older adult oncology population, addressing these conditions remains a priority in geriatric oncology research [12,14].

Studies examining frailty and pre-frailty in older adults with a history of cancer have been limited to outpatient settings [15]. Limited studies have determined the prevalence and outcomes of frailty depending on a history of cancer in acutely hospitalized older adults.

This study had two aims. First, to expand the limited knowledge of characteristics related to frailty and cancer by describing and comparing the frailty status and patient characteristics between older adults with a history of cancer and those without who are admitted to the acute medical unit (AMU) of a tertiary hospital. Second, to better understand the impact of a history of cancer on clinical outcomes during hospital stay by comparing hospital length of stay (LOS) and inpatient mortality between these two groups. We hypothesize that there will be a difference in frailty statuses between older adults with and without a history of cancer and that those with a history of cancer will have a poorer clinical outcome compared to those without.

## 2. Materials and Methods

### 2.1. Study Design and Setting

A prospective observational study was conducted in a cohort of hospitalized older adults ≥65 years (*n* = 329), admitted to the AMU, Flinders Medical Centre, Adelaide, South Australia. The AMU is a 30-bed medical unit that accepts patients who are referred directly from the emergency department for medical admission. This includes short-stay medical patients who require admission for ≤48 h and are discharged directly from the AMU. Patients may also be referred to long-stay medical wards from the AMU if they are deemed, by their attending physician, that they need further care after 48 h. The AMU also admits patients for the geriatric services of the hospital, including older patients who require input from the Older Persons Assessment Liaison team and nursing home patients who are seen by the Residential Care Outreach Service team.

### 2.2. Recruitment and Ethics

All eligible patients ≥65 years, admitted between February and September 2020 to the AMU, were approached for participation in this study within 48 h of their hospital admission. As many of the assessments were only available in English and had components that required patients to self-assess, patients who were unable to speak English, or those with cognitive impairment (Mini-Mental State Examination (MMSE) score ≤ 24), [16] and were unable to provide valid consent, were excluded. Patients who were under palliative care/severely or critically ill were deemed by the treating clinical team as unsafe to participate in this research study and were also excluded. This study was approved by the Southern Adelaide Clinical Human Research Ethics Committee (HREC reference number: HREC/19/SAC/240), within which the work was undertaken and conforms to the provisions of the Declaration of Helsinki in 1995 (as revised in Edinburgh 2000). Written informed consent was obtained from each participant. Participants were recruited by research staff not involved in their usual care. This study was reported in accordance with the Strengthening the Reporting of Observational Studies in Epidemiology (STROBE) guidelines [17].

### 2.3. Measures

#### 2.3.1. Frailty Status

The EFS was used to assess frailty status. The EFS assesses nine domains contributing to frailty–cognition, general health status, functional independence, social support, medication use, nutrition, mood, continence and functional performance [9]. The EFS score ranges from 0 to 17 points with higher scores indicative of a greater severity of frailty [6]. The total score categorizes the patient into one of the following five categories: not frail; pre-frail; mildly frail; moderately frail; and severely frail. For this study, we simplified the frailty categories by dividing the EFS score into three categories—robust (0–5), pre-frail (6–7) and frail (≥8)—as the degree of frailty was not a primary focus.

#### 2.3.2. History of Cancer

Information on the history of or existing cancer(s), type and current status(es) of cancer was collected from electronic medical records and patient case files at the time of recruitment.

#### 2.3.3. Other Patient Factors and Clinical Outcomes

The following patient factors of all participants were collected from electronic medical records and case files: age, sex, weight (kg), height (m), day of admission, comorbidities, number of medications, vitamin D supplementation, living status (living alone or with partner/friend), income and education level. The body mass index (BMI), kg/height (m^2^), was calculated from weight and height. The Mini-Mental State Examination (MMSE) was used to ascertain cognition [18]. A research dietitian conducted nutritional assessments on all participants using the PG-SGA and scored PG-SGA [19]. The Charlson Comorbidity Index (CCI) was used to assess the burden of comorbidities [20]. The clinical outcomes of interest for this study, hospital LOS and in-patient mortality, were also recorded.

### 2.4. Statistical Analysis

All statistical analyses were performed with SAS software version 9.3, IBM SPSS Statistics version 28.0 (IBM Corp, Armonk, NY, USA) and STATA version 18.0, and two-sided *p*-values < 0.05 were considered statistically significant. The sample size for this study was determined using a sample size calculator with the “CI for one proportion” method, based on a previously reported frailty prevalence of 0.7, and with an alpha level of 0.05. The sample size calculator from the Australian Bureau of Statistics was utilized, considering the population of older adults in South Australia in 2019 and prevalence data from two earlier studies [21,22,23]. Normality tests, one-way ANOVA, *t*-test and Chi-square tests of independence using a range of demographic and clinical variables compared “older adults with history of cancer” and “older adults without cancer”. Additional Kruskal–Wallis H tests were conducted for CCI. We used multivariable models for older adults with a history of cancer and those without to identify the characteristics associated with frailty and pre-frailty, with robust individuals serving as the reference group. The results were reported as odds ratio (OR) with 95% confidence interval (CI). Additionally, we employed multivariable Poisson regression, adjusting for various patient characteristics (age, sex, BMI, CCI, number of medications, use of vitamin D, living situation, education and income level and malnutrition status) to compare hospital LOS between older adults with and without a history of cancer, and the findings reported as an incidence rate ratio (IRR) with 95% CI.

## 3. Results

**Figure 1:**
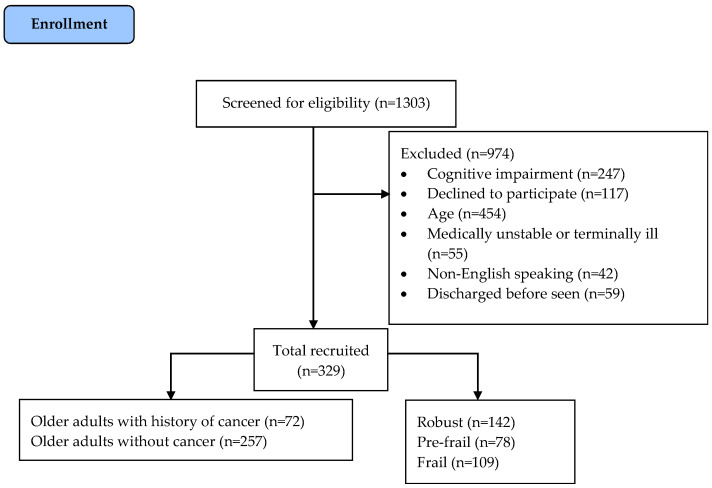
Flowchart of consecutive patient recruitment for present analyses.

Figure 1 illustrates the flow of patient recruitment wherein 329 consecutive older adult patients were included in the study. Normality tests revealed a normal distribution for all measures except for CCI and LOS.

### 3.1. Patient Characteristics

Characteristics of the study cohort organized by a history of cancer are presented in Table 1. Overall, 22% of hospitalized older adults (*n* = 72) had a history of cancer. The cancer types were prostate (*n* = 20), breast (*n* = 13), lung (*n* = 8), gastrointestinal (*n* = 8), skin (*n* = 6), colorectal (*n* = 5), head and neck (*n* = 2), liver (*n* = 3), ovarian (*n* = 2) and others (*n* = 4). Eight participants had cancers that were metastatic. There was no significant difference in the prevalence of pre-frailty/frailty among patients with or without a history of cancer (58% vs. 56%, respectively, *p* > 0.05). Older adults with a history of cancer were significantly more likely to be male, with a higher comorbidity burden, and poorer nutritional status when compared to those without a history of cancer (*p* < 0.05 for all).

### 3.2. Characteristics and Clinical Outcomes Associated with Frailty and Pre-Frailty

According to the multivariate model (Table 2) that included all participants with a history of cancer, pre-frailty/frailty demonstrated significant associations with both polypharmacy (OR 8.26, 95% CI: 1.74 to 39.2) and malnutrition (OR 8.91, 95% CI: 2.15 to 36.9). Conversely, among older adults without a history of cancer, pre-frailty/frailty was associated with a higher CCI (OR 2.64, 95% CI: 1.13 to 6.20) and a lower education level (OR 2.64, 95% CI: 1.13 to 6.20), in addition to polypharmacy (OR 2.98, 95% CI: 1.57 to 5.66) and malnutrition (OR 9.03, 95% CI: 4.25 to 19.2). In older adults with a history of cancer, while the median (IQR) hospital LOS was longer compared to those without cancer, the result was not statistically significant (3 (2, 6) versus 2 (2, 4) days, *p* = 0.085). Unadjusted analysis indicated that the risk of experiencing a prolonged LOS was 41% higher in older adults with a history of cancer than in those without (incidence risk ratio (IRR) 1.41, 95% CI 1.26 to 1.58, *p* < 0.001). Following adjusted analysis which accounted for factors such as age, gender, BMI, living status, Charlson index, vitamin D supplementation, SGA status, income level, prior admissions and EFS score, the LOS remained significantly longer in older adults with a history of cancer than in those without (IRR 1.24, 95% CI 1.10 to 1.41, *p* < 0.001). In-patient mortality was not analyzed due to the occurrence of only one death during hospitalization.

## 4. Discussion

Our study found more than one-fifth of hospitalized older adults in the AMU had a history of cancer. The prevalence of pre-frailty and frailty amongst them were comparable to those without a history of cancer. Regardless of cancer history, pre-frailty/frailty was independently associated with polypharmacy and malnutrition. However, the risk of having a prolonged LOS was 24% higher in older adults with a history of cancer than those without, prompting further investigations for systematic assessment and timely interventions for older adults with a history of cancer admitted to the AMU.

The results of this study concur with a recent review of 20 studies evaluating frailty and pre-frailty in older cancer patients (inpatient, hospital outpatient and community settings), which revealed that frailty/pre-frailty affected more than half of patients (median prevalence of 68%; range of 12% to 89%) [15]. Although frailty is prevalent across hospital wards, its distribution varies, contingent upon factors such as ward type, whether medical or surgical, which may account for the diversity in prevalence rates. Findings from this study support routine screening and assessment of frailty and pre-frailty in older adults admitted to the AMU, including those with a history of cancer, to inform decisions on treatment and intervention.

Findings from the present study also highlight polypharmacy and malnutrition as two important factors associated with pre-frailty/frailty in older inpatients, regardless of their cancer history, aligning with previous research in acute care and community settings [24,25]. These results have implications for clinical practice, especially in the AMU. Pre-frail and frail older adults often have several co-existing chronic conditions that require pharmacological management, leading to multiple medications being prescribed. Given the significance of polypharmacy in this group of patients, the establishment of a medication reconciliation service at the AMU could foster better communication between pharmacists and physicians. This initiative has the potential not only to deprescribe unnecessary medications but also to mitigate medication errors and ultimately reduce hospital LOS [26].

Likewise, malnutrition is still often missed in hospital settings due to suboptimal nutrition screening practices in hospitalized older adults, including those with cancers [27]. To alleviate malnutrition, strategies to prevent and treat frailty and pre-frailty can consider services to implement dietary change [28]. Referrals for such nutritional interventions should also be coupled with exercise to improve frailty and frailty-related indicators in hospitalized older adults [29,30]. While it is important to treat malnutrition during hospitalization, efforts should also target prevention and early intervention at the primary care level through multidisciplinary teams involving exercise health professionals and dietitians [31,32]. Whilst acknowledging the increasing burden of geriatric and oncological care on general practitioners in primary care settings, a recent novel study provided preliminary evidence that dietitians could potentially act as first contact practitioners for older adults at risk of malnutrition (and frailty) as part of the practice multidisciplinary team [33].

Further to their association with frailty and pre-frailty, polypharmacy and malnutrition can be interrelated. A recent review demonstrated a significant association between polypharmacy (defined as the use of five or more medications) and malnutrition [34]. The use of drugs is associated with a number of side effects, some of which can impact nutritional intake such as nausea, decreased appetite and decreased saliva production [35]. While malnutrition may not be a direct causal factor for more medication, patients who are malnourished have a higher risk of adverse events which may require additional medications as part of its management. For example, post-operative infections may require antibiotic treatment, or individuals undergoing cancer therapy may experience more pronounced side effects, leading to the need for additional medications [36].

The median LOS found in this study from the AMU corresponds with and adds to reports from other studies: a cohort study of hospitalization in cancer patients (i.e., cancer types: breast, prostate, lung, gastrointestinal and colorectal) with LOS of the median (IQR) of 3 (2–7) days, and another retrospective study of in-hospital LOS after surgical oncological procedures with LOS of median (IQR) of 4 (2–7) days [37,38]. Furthermore, similar to another study, a comparison between cancer and non-cancer diagnoses has shown that hospital LOS was longer for patients with cancer [39].

There could be several reasons for older adults with a history of cancer have a longer hospital LOS compared to their non-cancer counterparts. Older adults with active cancers deal with acute side effects of treatment or cancer itself, while those that survive have to cope with long-term and/or late effects of treatments—physical and psychological issues, e.g., lymphoedema, osteoporosis, pain, fatigue, altered sleep and cognition [40,41]. Consequently, admission to an acute care setting can sometimes exacerbate these symptoms and could trigger a cascade of additional investigations that may not be directly related to the presenting problem. In addition, cancer care often involves input from a multidisciplinary team of specialists including oncologists, nurses and allied health professionals, and delays in organizing care among these different specialties may extend the hospital stay [42]. It is worth highlighting that, in this cohort, 11% of the older adults with a history of cancer were living with metastatic cancers. Future research could prioritize enhancing access to specialist advice and triage services within the community settings. By doing so, we can proactively address the intricate needs of older adults with a history of cancer, intervening before these needs escalate to emergency department visits or inpatient hospitalizations [43].

Several strategies have been proposed to bridge existing health services across settings for older adults with cancer so these services can better meet their complex needs. Montroni and colleagues proposed a six-step multidimensional, multiphase pathway to optimize the care of older adults with cancer undergoing surgery that included co-management in the long term, as recovery continues after hospital discharge, e.g., linking operative service with prehabilitation and transitional care management [44]. Another strategy, which is probably more achievable within the community, is the integration of comprehensive geriatric assessment into community oncology practices, which has been associated with improved patient-centered communication, reduced hospital LOS and treatment toxicity in older adults with cancer [45,46].

While a longer LOS may seem burdensome, it can serve as an opportunity for beneficial interventions, especially in older adults with complex health needs. Unplanned hospitalization can be an opportunity in disguise to allow for conducting geriatric and frailty assessments, which can help identify gaps in care and pinpoint areas for improvement. These assessments may help healthcare professionals tailor treatment plans and make referrals to appropriate services, ultimately improving outcomes for older patients [47].

This study has several strengths. Frailty and pre-frailty were measured with a validated assessment tool in older adults with and without cancer that incorporates the multidimensional domains (cognition, general health status, functional independence and performance, social support, medication use, nutrition, mood, continence) included in a comprehensive geriatric assessment [48,49,50]. The consecutive sampling also reduced bias in the selection of participants during the data collection period. However, this study is not without limitations. A sensitivity analysis replacing frailty defined by EFS with another frailty assessment tool could not be conducted, as frailty was not concurrently measured with another validated tool. Future studies can consider measuring frailty with different tools to assess the relationship between frailty status and LOS and other outcome measures. Participants in this study may not represent older adults who were unable to speak in English, with mild to severe cognitive impairments, or those directly admitted to general wards from the emergency department. Therefore, the generalizability of the results to other populations or settings may be limited. Lastly, inpatient mortality could not be examined due to the lack of incidences reported within this study period.

## 5. Conclusions

The prevalence of frailty/pre-frailty did not differ between older adults with a history of cancer and those without in the AMU. Factors associated with frailty/pre-frailty, regardless of history of cancer, are not different from those reported in other settings; polypharmacy and malnutrition are two important factors in both older adults with and without a history of cancer. Acutely hospitalized older adults with a history of cancer had a higher risk of a longer LOS compared to those without cancer. This prompts further investigations not only into systematic assessment to identify those at risk but also into trialing new models of care or evolving existing health services to provide early interventions that extend to the community to meet the complex needs of this vulnerable population.

## Figures and Tables

**Table 1:** Characteristics of cohort by history of cancer (*n* = 329).

	History of Cancer (*n* = 72)	Without Cancer (*n* = 257)	*p*-Value ^a^
Age ^b^	79.7 ± 6.5	78.9 ± 8.6	0.483
Age (years) ^c^			0.773
65–79	37 (51%)	137 (53%)	
≥80	35 (49%)	120 (47%)	
BMI ^b^	26.4 ± 5.4	27.1 ± 6.5	0.396
BMI ^c^			0.738
Under/healthy weight	31 (43%)	105 (41%)	
Overweight/obese	41 (57%)	153 (59%)	
Sex ^c^			<0.001
Male	46 (64%)	105 (41%)	
Female	26 (36%)	152 (59%)	
CCI score ^b^	4.9 ± 2.0	4.3 ± 1.3	0.003
CCI score ^c^			0.556
0–3	19 (26%)	77 (30%)	
≥4	53 (74%)	180 (70%)	
No. of medications ^b^	6.8 ± 3.7	6.1 ± 3.6	0.155
No. of medications ^c^			0.790
0–4	24 (33%)	90 (35%)	
≥5	48 (67%)	167 (65%)	
On vitamin D ^c^			0.275
Yes	20 (28%)	89 (35%)	
No	52 (73%)	168 (65%)	
Living alone ^c^			0.065
Yes	24 (33%)	117 (46%)	
No	48 (68%)	140 (54%)	
Education level ^c^			0.844
Tertiary	31 (43%)	114 (44%)	
Up to secondary	41 (57%)	143 (56%)	
Income level ^c^			0.166
≤20 k	36 (50%)	105 (41%)	
>20 k	36 (50%)	152 (59%)	
PG-SGA ^c^			0.001
Well nourished	34 (47%)	175 (68%)	
Malnourished	38 (53%)	82 (32%)	
EFS category ^c^			0.772
Robust	30 (42%)	112 (44%)	
Pre-frail/Frail	42 (58%)	145 (56%)	

BMI: Body Mass Index; CCI: Charlson comorbidity index; PG-SGA: Patient-generated Subjective Global Assessment; EFS: Edmonton Frail Scale; ^a^ *p*-values obtained by one-way ANOVA or *t*-tests for continuous variables and Chi-square tests for categorical variables; ^b^ values reflect the mean (standard deviation) for continuous variables; ^c^ values expressed as absolute numbers (percentage) for categorical variables; percentage may not add up due to rounding.

**Table 2:** Multivariate models examining the characteristics associated with pre-frailty/frailty status (versus robust) in older adults with a history of cancer and those without cancer.

Characteristics	History of Cancer OR (95% CI)	Without Cancer OR (95% CI)
Age		
65–79	Reference	Reference
≥80	0.88 (0.18–4.39)	1.60 (0.77–3.36)
Sex		
Male	Reference	Reference
Female	1.51 (0.35–6.55)	1.04 (0.55–1.95)
BMI		
Underweight/healthy weight	Reference	Reference
Overweight/obese	3.03 (0.76–12.03)	1.84 (0.98–3.44)
CCI score		
0–3	Reference	Reference
≥4	0.91 (0.14–5.90)	2.64 (1.13–6.20) *
No. of medications		
0–4	Reference	Reference
≥5	8.26 (1.74–39.16) *	2.98 (1.57–5.66) *
On vitamin D		
Yes	Reference	Reference
No	0.68 (0.14–3.38)	1.36 (0.72–2.57)
Living alone		
No	Reference	Reference
Yes	1.76 (0.39–7.96)	1.09 (0.59–2.01)
Education level		
Tertiary	Reference	Reference
Up to secondary	1.78 (0.47–6.73)	2.14 (1.17–3.91) *
Income level		
>20 k	Reference	Reference
≤20 k	0.29 (0.07–1.17)	1.18 (0.64–2.18)
PG-SGA		
Well nourished	Reference	Reference
Malnourished/malnutrition	8.91 (2.15–36.89) *	9.03 (4.25–19.20) *

OR: odds ratio; CI: confidence interval; BMI: Body Mass Index; CCI: Charlson comorbidity index; PG-SGA: Patient-generated Subjective Global Assessment. * *p* < 0.05.

## Data Availability

The raw data supporting the conclusions of this article will be made available by the authors upon request.
